# Side Effects of COVID-19 Vaccines (Pfizer, AstraZeneca) in Saudi Arabia, Eastern Province

**DOI:** 10.7759/cureus.27297

**Published:** 2022-07-26

**Authors:** Fatma Hamza Ibrahim Amer, Remah Alzayyat, Nof Alzayyat, Sarh Alomran, Sumaya Wafai, Haila Alabssi, Deem Alsultan

**Affiliations:** 1 Family and Community Medicine, Imam Abdulrahman Bin Faisal University, Dammam, SAU; 2 Family and Community Medicine, Arabian Gulf University, Manama, BHR

**Keywords:** coronavirus disease, post covid-19 vaccination, astrazeneca vaccine, pandemic, vaccination, saudi arabia, pfizer vaccine, covid-19, covid-19 side effects, covid-19 vaccine

## Abstract

Background: The coronavirus disease 2019 (COVID-19) virus has wreaked havoc on the planet, causing death and illness. Effective vaccination to eradicate the virus is the best approach to safeguarding the globe from it. Our study is considered one of the earliest studies conducted to determine the side effects of COVID-19 vaccines. We started data collection from May 2021 till September 2021, which was the beginning period of vaccine distribution in Saudi Arabia. This study aims to look at potential side effects and factors that contribute to their occurrence.

Methods: The optimal study design for achieving our goals was survey-based. Following Institutional Review Board approval, we created an online self-administered questionnaire using the Google survey webpage (Google LLC, Mountain View, California, United States). We disseminated the survey to 2293 individuals from May 2021 till September 2021 in the eastern province of Saudi Arabia, to males and females above the age of 18 who have been vaccinated by either Pfizer or AstraZeneca in one dose or two doses.

Results: The most prevalent side effect was pain at the injection site (60.7%), followed by general fatigue (23.8%) and swelling at the injection site (16.7%), with shortness of breath being the least common (0.9%). When the prevalence of COVID-19 vaccine side effects was compared to the socio-demographic characteristics of participants, we discovered that those without associated comorbidity (p=0.025) and non-smoking participants (p=0.009) showed more side effects. On the other hand, those who received Pfizer vaccine (p0.001) and those who exercised regularly (p0.001) had lower rates of COVID-19 vaccine side effects. Also, obesity was shown to be the most commonly related disease in terms of comorbidities (8.5%), followed by allergy (4.9%) and asthma (4.6%).

Conclusion: We find that vaccination against COVID-19 has only minor adverse effects. Therefore we anticipate that this study will assist in dispelling rumors about dangerous side effects of the COVID-19 vaccine.

## Introduction

Coronavirus disease (COVID-19) is a novel disease that caused a pandemic and there has been no doubt that vaccination is the most effective way to combat it. Therefore, as a developed country, Saudi Arabia made vaccination mandatory to minimize or eradicate COVID-19. Within a year, a few COVID-19 vaccines were developed and authorized. Due to the rapid development of the vaccine and minimum time to test it, multiple assumptions and claims about possible side effects and complications appeared against it.

Although it is valid to assume that any vaccine has risks, according to the Centers for Disease Control and Prevention (CDC), severe adverse events are rare, and most minor side effects resolve within a few days [1]. Possible systemic side effects include fatigue, generalized pain, headaches, chills, and fever. Injection site side effects include pain, redness, and swelling [1]. Furthermore, the United States Food and Drug Administration (FDA) ensures that vaccination of COVID-19 will decrease the severity of coronavirus disease by 50% [2].

We conducted a cross-sectional survey-based study, which is one of the first exploratory studies done at the start of vaccination campaigns in Saudi Arabia. Despite the growing literature on the topic, our study is the first to be done in the Eastern Province, adding to the existing literature and gathering national data on real-world COVID-19 side effects reported by the public. The study aims to detect, confirm, or deny the possible side effects of COVID-19 vaccines (Pfizer, AstraZeneca) in Saudi Arabia, Eastern Province, from January 2021 to December 2021 among the general Saudi population. 

## Materials and methods

Study design

This is an online survey-based study conducted in Saudi Arabia, Eastern Province. We conducted the study in 2021, between January and December. Data collection took place between May 2021 and September 2021. There were a total of 2293 responses. However, we excluded 207 responses due to insufficient data. The final analysis involved 2086 responses that had all of the necessary details.

Ethical clearance

This study was carried out after gaining ethical approval from the Imam Abdulrahman bin Faisal University Institutional Review Board (IRB), Dammam, Saudi Arabia, dated April 18, 2021 (approval number IRB-2021-01-174). Getting the participants' consent was the first step in the data collection process. When a participant first clicked the link to the survey, an informed consent form appeared. We provided the participants with information regarding the objectives and rationale of the study as part of the informed consent procedure. We informed all participants that their names would be kept anonymous and their data would be kept private.

Study tool

We distributed the questionnaire to the general public using the Google survey webpage (Google LLC, Mountain View, California, United States). The questionnaire was in Arabic and English and contained 45 questions, divided into four parts. The first part was about demographic data (gender, age, date of birth, job, level of education, weight, and height). The second part aimed to gather information on the participant's health status (chronic illness presence, if taking any medications). The third part was about the participant's habitual behaviors (exercising, diet, smoking, alcohol, and illegal drug use). The last part was regarding the COVID-19 status of the participant (prior infection with the virus, intensive care unit (ICU) admissions, having taken the vaccine, type of vaccine taken, any side effects of vaccine).

Questionnaire validation

The questionnaire was validated through expert reviews and pilot studies. The department's expert committee gave its approval to the questionnaires. The committee consists of subject matter experts with training in both English and Arabic, as well as survey analysis. They assessed the questions' phrasing, topics, application to each field, and grading procedures. We did the pilot study on 30 carefully chosen volunteers of various ages, genders, professions, and educational levels. A committee of experts analyzed their comments and made necessary adjustments before the survey was published online for data collection. The results of the pilot study were not taken into account in the final analysis of the research results because several questions were inconsistent.

Inclusion and exclusion criteria

Any Saudi Arabian resident above the age of 18 who had been vaccinated by either Pfizer or AstraZeneca in one dose or two doses, had internet access, and had a willingness to participate in the study met the inclusion criteria. We excluded participants under the age of 18 and those who were not vaccinated with even a single dose. Participants who could not read Arabic or English, had no internet connection, or did not have the required technical skills were excluded. Participants were not individually approached or offered anything in exchange for participating in the study. Non-completion of survey responses was not taken into consideration while analyzing the results.

Statistical analysis

IBM SPSS Statistics for Windows, Version 26.0 (Released 2019; IBM Corp., Armonk, New York, United States) was used to analyze the data. We used the Chi-square test to investigate the relationship between COVID- 19 vaccination side effects and socio-demographic factors. The significant data were then entered into a multivariate regression model to establish the independent significant factor related to the risk of having a COVID-19 vaccination side effect, along with an adjusted odds ratio and a 95% confidence interval. The significance threshold of P=0.05 was used as the cut-off value.

## Results

In total, 2086 individuals met the inclusion criteria. Table 1 presents the socio-demographic characteristics of the participants. The most common age group was 18-25 years (61.6%), with the majority being females (67.5%). Participants who were students constituted 29.3% while medical sector employees constituted 23.5%. Furthermore, the majority of the respondents held university degrees (70.9%). Regarding body mass index (BMI), 45.1% had a normal BMI while 26.1% were overweight. The proportion of participants who were regularly taking medicine was 21.9%. In addition, 17.9% exercised twice a week and 15.3% exercised daily.

**Table 1:** Socio-demographic characteristics of participants

Characteristics	N (%)
Age group	
18 – 25 years	1284 (61.6%)
26 – 35 years	255 (12.2%)
36 – 45 years	259 (12.4%)
46 – 55 years	204 (09.8%)
56 – 65 years	71 (03.4%)
>65 years	13 (0.60%)
Gender	
Male	678 (32.5%)
Female	1408 (67.5%)
Occupational status	
Employed in the medical sector	490 (23.5%)
Employed in the military sector	14 (0.70%)
Employed in the public sector	338 (16.2%)
Employed in the private sector	145 (07.0%)
Student	612 (29.3%)
Retired	68 (03.3%)
Housewife	163 (07.8%)
Unemployed	256 (12.3%)
Educational level	
Less than secondary school	21 (01.0%)
Secondary	458 (22.0%)
University	1480 (70.9%)
Master degree	93 (04.5%)
PhD	34 (01.6%)
BMI Level	
Underweight (<18.5 kg/m2)	191 (09.2%)
Normal (15.5 – 24.9 kg/m2)	941 (45.1%)
Overweight (25 – 29.9 kg/m2)	544 (26.1%)
Obese (≥30 kg/m2)	410 (19.7%)
Taking medicine regularly	
Yes	456 (21.9%)
No	1630 (78.1%)
Having exercise	
Daily	319 (15.3%)
Twice a week	373 (17.9%)
Once a week	273 (13.1%)
Monthly	256 (12.3%)
I don’t do exercise	780 (37.4%)
Other	85 (04.1%)

**Figure 1:**
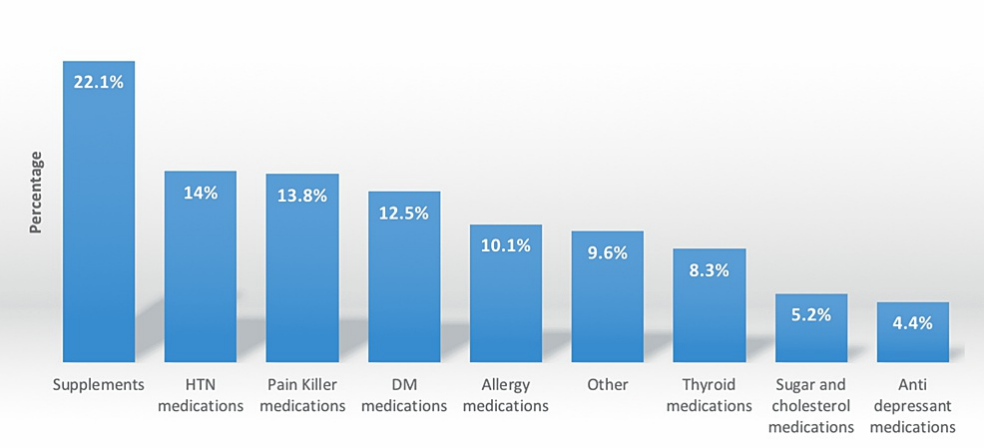
Type of medication use HTN: hypertension; DM: diabetes mellitus

In Figure 1, it is seen that the most frequently used medication was supplements (22.1%), followed by hypertension medications (14%) and painkillers (13.8%), while antidepressant medication was the least (4.4%).

**Figure 2:**
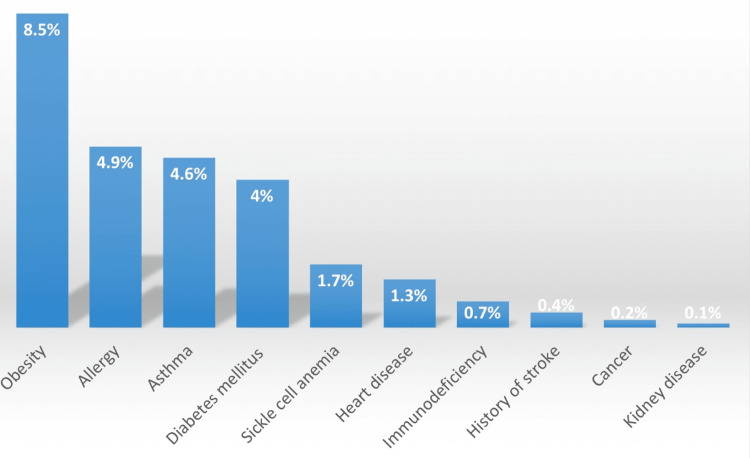
Associated comorbidity

Figure 2 shows the associated comorbidity of participants who received COVID-19 vaccination. We observed that obesity was the most commonly associated disease (8.5%) followed by allergy (4.9%) and asthma (4.6%).

**Figure 3:**
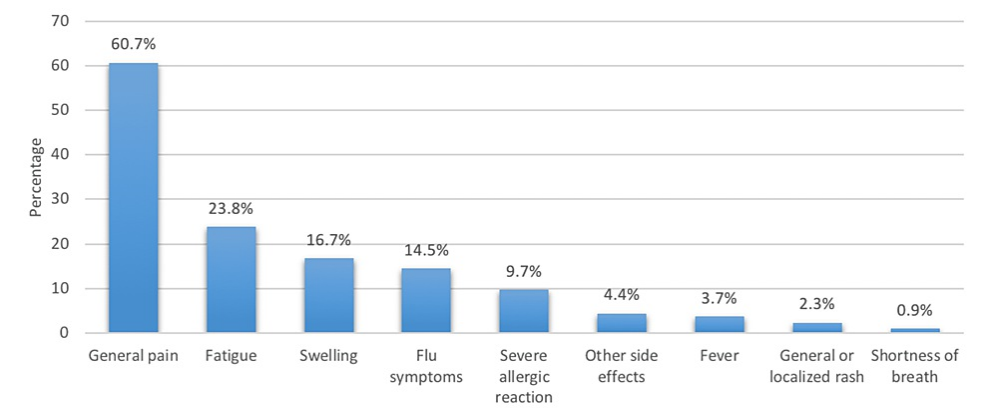
COVID-19 vaccine side effects COVID-19: coronavirus disease 2019

Figure 3 depicts the COVID-19 vaccine side effects. It revealed that the most commonly experienced side effect was general pain (60.7%), followed by fatigue (23.8%) and swelling (16.7%), while shortness of breath was the least common (0.9%). Other side effects reported by participants include headaches, dizziness, changes in the menstrual cycle, joint pain, abdominal pain, shivering, and palpitations.

The characteristics of participants who received the COVID-19 vaccine are described in Table 2. Based on the results, 36.3% expressed that they have a balanced diet. Participants who smoke and drink alcohol regularly constitute 7.4% and 0.1% of the sample, respectively. This study also noted that 1.3% took medication without a proper prescription. The prevalence of participants who had been infected by COVID-19 was 14.7%. Of them, 2.9% required intensive care. Furthermore, the majority of participants (76.2%) received the Pfizer vaccine. More than half (54%) received two doses, whether from Pfizer or Astra-Zeneca. The prevalence of respondents who experienced side effects after taking the vaccination was 83.1%.

**Table 2:** Characteristics of participants who had been vaccinated against COVID-19 COVID-19: coronavirus disease 2019

Variables	N (%)
Is your food balanced with health?	
Yes	758 (36.3%)
No	1187 (56.9%)
I don’t know	141 (06.8%)
Smoking	
Yes, daily	154 (07.4%)
Yes, sometimes	138 (06.6%)
No	1794 (86.0%)
Alcohol	
Yes, daily	03 (0.10%)
Yes, sometimes	13 (0.60%)
No	2070 (99.2%)
Taking medicine without a prescription	
Yes, daily	28 (01.3%)
Yes, sometimes	603 (28.9%)
No	1455 (69.8%)
Have you had COVID-19?	
Yes	307 (14.7%)
No	1779 (85.3%)
Required artificial respiration/Intensive care after COVID-19 infection ^(n=307)^	
Yes	09 (02.9%)
No	298 (97.1%)
Type of vaccine	
Pfizer	1590 (76.2%)
Astra-Zeneca	422 (20.2%)
Both	74 (03.5%)
Number of doses	
Single	960 (46.0%)
Two doses	1126 (54.0%)
Suffer side effects after taking the vaccine	
Yes	1733 (83.1%)
No	353 (16.9%)

When measuring the impact of COVID-19 vaccine side effects in regards to the socio-demographic characteristics of participants, we found that the prevalence of COVID-19 vaccine side effects was more common among those in the younger age group (p<0.001), female gender (p<0.001), students (p=0.001), those with normal BMI (p<0.001), those without associated comorbidity (p=0.025), and non-smoking participants (p=0.009). In contrast, it was less common among those exercising regularly (p<0.001) and those who received the Pfizer vaccine (p<0.001) (Table 3).

**Table 3:** Association between COVID-19 vaccine side effects and socio-demographic characteristics ^* ^P-value has been calculated using Chi-square test; ** Significant at p<0.01 level COVID-19: coronavirus disease 2019

Factor	Suffered COVID 19 vaccine Side Effect		P-value *
	Yes N (%) ^(n=1733)^	No N (%) ^(n=353)^	
Age group			
≤25 years	1114 (86.8%)	170 (13.2%)	<0.001 **
>25 years	619 (77.2%)	183 (22.8%)	
Gender			
Male	492 (72.6%)	186 (27.4%)	<0.001 **
Female	1241 (88.1%)	167 (11.9%)	
Occupational status			
Employed	823 (83.4%)	164 (16.6%)	0.001 **
Unemployed	381 (78.2%)	106 (21.8%)	
Student	529 (86.4%)	83 (13.6%)	
Educational level			
Secondary or below	394 (82.3%)	85 (17.7%)	0.584
University or higher	1339 (83.3%)	268 (16.7%)	
BMI Level			
Normal or underweight	977 (86.3%)	155 (13.7%)	<0.001 **
Overweight or obese	756 (79.2%)	198 (20.8%)	
Associated comorbidity			
Yes	363 (79.6%)	93 (20.4%)	0.025 **
No	1370 (84.0%)	260 (16.0%)	
Having exercise			
Yes	1055 (80.8%)	251 (19.2%)	<0.001 **
No	678 (86.9%)	102 (13.1%)	
Smoking			
Yes	227 (77.7%)	65 (22.3%)	0.009 **
No	1506 (83.9%)	288 (16.1%)	
Having balanced health with food			
Yes	612 (80.7%)	146 (19.3%)	0.096
No	1001 (84.3%)	186 (15.7%)	
I don’t know	120 (85.1%)	21 (14.9%)	
Have you had COVID-19			
Yes	257 (83.7%)	50 (16.3%)	0.748
No	1476 (83.0%)	303 (17.0%)	
Type of Vaccine			
Pfizer	1282 (80.6%)	308 (19.4%)	<0.001 **
Astra-Zeneca	381 (90.3%)	41 (09.7%)	
Both	70 (94.6%)	04 (05.4%)	
Number of doses			
Single dose	799 (83.2%)	161 (16.8%)	0.865
Two doses	934 (82.9%)	192 (17.1%)	

We then performed a multivariate regression analysis to determine the independent significant predictor associated with the risk of COVID-19 vaccine side effects. We noted that female gender and Astra-Zeneca vaccine to be the independent significant factors associated with increased risk of COVID-19 vaccine side effects. At the same time, being in the older age group (>25 years) and having regular exercise were the independent significant factors associated with a decreased risk of COVID-19 vaccine side effects, showing that those in the older age group (adjusted odds ratio (AOR)=0.721; 95% CI=0.549-0.947; p=0.019) and those who exercise regularly (AOR=0.717; 95% CI=0.553-0.931; p=0.013) had a 30% lower risk of COVID-19 vaccine side effects. On the other hand, the risk of females having COVID-19 vaccine side effects was twice as high as that of males (AOR=2.660; 95% CI=2.038-1.449; p<0.001). Similarly, patients who received the AstraZeneca vaccine were four times more associated with the risk of COVID-19 vaccine side effects than those who received the Pfizer vaccine (AOR=3.966; 95% CI=1.421-11.068; p=0.009). Other variables included in the model, such as occupational status, BMI level, associated comorbidity, and smoking did not show a significant effect after adjustment to the regression model (p>0.05) (Table 4).

**Table 4:** Multivariate regression analysis to determine the association of socio-demographic characteristics with having COVID-19 vaccine side effects ** Significant at p<0.01 level BMI: body mass index; AOR: adjusted odds ratio; CI: confidence interval

Factor	AOR	95% CI	P-value
Age group			
≤25 years	Ref		
>25 years	0.721	0.549 – 0.947	0.019 **
Gender			
Male	Ref		
Female	2.660	2.038 – 3.471	<0.001 **
Occupational status			
Employed	Ref		
Unemployed	1.069	0.789 – 1.449	0.665
Student	1.387	0.980 – 1.963	0.065
BMI Level			
Normal or underweight	Ref		
Overweight or obese	0.848	0.651 – 1.104	0.220
Associated comorbidity			
Yes	1.138	0.857 – 1.510	0.372
No	Ref		
Having exercise			
Yes	0.717	0.553 – 0.931	0.013 **
No	Ref		
Smoking			
Yes	1.270	0.902 – 1.788	0.171
No	Ref		
Type of Vaccine			
Pfizer	Ref		
Astra-Zeneca	3.966	1.421 – 11.068	0.009 **
Both	1.631	0.559 – 4.758	0.371

The results of the study show that the most prevalent side effect is pain at the injection site, followed by general fatigue and swelling at the injection site, with shortness of breath being the least common. Also, we discovered that those who exercised regularly and those who received the Pfizer vaccine had lower rates of COVID-19 vaccine side effects. On the other hand, side effects from the COVID-19 vaccine were more likely in people who did not have any comorbidities, did not smoke, were females, or had a normal BMI. The results also showed that obesity, followed by allergies and asthma, were the most common comorbidities. 

## Discussion

This study highlights post-vaccination side effects associated with Pfizer and AstraZeneca vaccines among individuals in the Eastern Province of Saudi Arabia. The frequent side effects identified in our study are not worrisome and include general pain, fatigue, flu-like symptoms, fever, injection site pain, and swelling. The CDC and the Saudi Ministry of Health confirmed these mild side effects in January of 2021 [1,3]. A randomized control trial by Polack et al. reported similar side effects and observed a low incidence of serious side effects [4].

The results of this study revealed that the most frequently experienced side effects were general pain, fatigue, and swelling. This finding is consistent with what Alhowaymel et al. reported in their study done in the northwestern Riyadh Province in Saudi Arabia [5]. Furthermore, we found shortness of breath to be the least common side effect following the COVID-19 vaccination. Similar results were reported by El-Shitany et al. [6].

This study also observed a higher frequency of COVID-19 vaccine side effects among younger people. This finding is likely because most participants were in the age group of 18-25 years. Sampling biases based on age could have occurred as older individuals are less likely to have Internet access or computer skills. However, comparable studies link this finding to the more robust immune response and more pronounced cytokine production in younger individuals compared to older individuals [6,7]. El-Shitany et al. observed that those under 60 experienced more flu-like symptoms [6]. Another similar result is reported by Alghamdi et al., where adults younger than 50 had a higher incidence and intensity of COVID-19 post-vaccination symptoms [7].

The results also showed a higher prevalence of side effects among female participants. Similarly, three other studies reported findings of females being more prone to COVID-19 side effects and attributed this to the variation in immune response between genders [8-9]. However, Alhowaymel et al.'s results were inconsistent with ours since they found that males are more likely to experience COVID-19 post-vaccination side effects [5].

The present study noted that non-smoking participants more frequently reported side effects. We could attribute this result to the small percentage (7.4%) of smokers in our sample. A study by Almughais et al. showed inconsistent results, as smokers had a higher frequency of post-vaccination side effects in their study [8].

Regarding vaccine type, patients who received the AstraZeneca vaccine had four times the risk of developing COVID-19 vaccine side effects than those who received the Pfizer vaccine. Comparable results were reported in two different articles by Almughais et al. and Alzarea et al. [8-9].

Study limitations

The findings of this study contribute to the national data by providing real-world, individual-reported data on COVID-19 vaccine side effects. However, when interpreting our findings, some limitations should be considered. First, we refer to specific side effects in the distributed questionnaire. However, due to the short time it took to make the vaccine and verify its efficacy, there may be other undiscovered adverse effects. Also, only two types of accessible vaccines in Saudi Arabia were studied. Recall bias is possible because some people reported the symptoms long after receiving the vaccine. The online distribution of the survey might allow selection bias. However, we attempted to mitigate this by reaching out to people in multiple settings, including shopping malls, clinics, family and friend gatherings, and social media platforms.

## Conclusions

The study demonstrates the possible side effects of the COVID-19 vaccines administered in Saudi Arabia, Eastern Province. It considers different factors that can play a role in developing side effects. According to our findings, vaccination against COVID-19 has modest side effects. As a result, we believe this research will help confirm minor side effects and debunk rumors regarding dangerous COVID-19 vaccination side effects and mitigate any remaining hesitancy toward receiving the vaccine.
